# Patients with multiple myeloma referred for palliative care consultation: from retrospective analysis to future directions to improve clinical outcomes

**DOI:** 10.1007/s00520-021-06560-8

**Published:** 2021-10-31

**Authors:** Maria Caterina Pallotti, Romina Rossi, Emanuela Scarpi, Monia Dall’Agata, Marianna Ricci, Michela Ceccolini, Sonia Ronconi, Vanessa Valenti, Marco Maltoni, Giovanni Martinelli, Claudio Cerchione

**Affiliations:** 1 Palliative Care Unit, IRCCS Istituto Romagnolo Per Lo Studio Dei Tumori (IRST), Dino Amadori”, Via P. Maroncelli 40, 47014 Meldola, FC Italy; 2 Unit of Biostatistics and Clinical Trials, IRCCS Istituto Romagnolo Per Lo Studio Dei Tumori (IRST) “Dino Amadori”, Meldola, FC Italy; 3 Hematology Unit, IRCCS Istituto Romagnolo Per Lo Studio Dei Tumori (IRST) “Dino Amadori”, Meldola, FC Italy; 4 Medical Oncology Unit, IRCCS Azienda Ospedaliero-Universitaria Bologna, Bologna, Italy; 5 Department of Specialized, Experimental and Diagnostic Medicine, University of Bologna, Bologna, Italy; 6 Scientific Directorate, IRCCS Istituto Romagnolo per lo Studio dei Tumori (IRST) “Dino Amadori”, Meldola, FC Italy

**Keywords:** Multiple myeloma, Pain, Early palliative care, Symptoms, Hospice, Homecare

## Abstract

**Introduction:**

New treatments have improved the overall survival of patients with multiple myeloma (MM). At diagnosis and during the course of the disease, patients often report pain and other symptoms. Given the long disease trajectory, psychological and social issues are also frequent. Recently, the potential usefulness of early palliative care (EPC) was hypothesized in the area of hematology. We conducted a retrospective analysis of patients with MM referred to our institute for a palliative care (PC) consultation between January 2017 and June 2020. Our aim was to evaluate the main reasons (pain or other clinical symptoms) for the referral for a first PC consultation.

**Methods:**

We examined the main reasons for the first PC consultation, the number of PC consultations carried out, and the period of time between diagnosis, first and subsequent PC visits, and death. We also recorded information on the type of pain experienced and the treatments administered.

**Results:**

Of the 325 patients with MM followed at our hematology unit during the study period, 43 were referred for a PC consultation (39 for pain management and 4 to determine the most appropriate care setting (hospice or palliative homecare service)). Nineteen (44.2%) of the 43 patients reported other symptoms in addition to pain. The median time between MM diagnosis and the first PC consultation was 473 days. Fifteen patients died, with a median 332 days between the first PC visit and death.

**Conclusion:**

Randomized studies on MM involving larger patient populations with access to EPC are needed to identify an effective clinical model to improve the management of patients with MM.

## Introduction

Multiple myeloma (MM) is the 14th most common type of cancer worldwide and represents 1.8% of all cancers in the USA. It is estimated that in 2020, there were 32,270 new cases and 12,830 deaths from this disease. It is more frequent in males, and the median age at diagnosis for both sexes is 69 years [1].

MM is characterized by an increase in plasma cells, resulting in an excessive production of immunoglobulin proteins with different signs and symptoms due to ensuing multiple organ damage, e.g., anemia from abnormal bone marrow involvement, osteolytic bone lesions, hypercalcemia and other skeletal-related events (SREs) from bone damage, and renal failure from kidney problems [2, 3].

More than two-thirds of patients at diagnosis and nearly all during the course of the disease have myeloma bone damage and experience pain from osteolytic bone lesions, but different types of pain occur at different stages of the disease. In fact, if bone pain is more frequent at diagnosis and relapse, pain from chemotherapy-induced peripheral neuropathy develops during MM treatment (especially bortezomib, thalidomide, lenalidomide, and vinca alkaloids) and can worsen over time from the prolonged use of these drugs. Oropharyngeal mucositis and somatic pain caused by the use of growth factors are frequent during stem cell transplantation, after which post-herpetic neuralgia is often diagnosed. Furthermore, long survivors may experience late effects of treatments such as chronic pain, neuropathy, or asthenia, and may also develop psychological symptoms such as depression and anxiety [2–6].

Each type of pain requires careful evaluation of the symptom (i.e., cause, localization, type, intensity) to identify the most effective treatment. The management of MM bone pain follows a multidisciplinary approach involving not only the specialized PC team, but also an orthopedic specialist/physician and radiotherapist. Analgesic therapy consists of opioids associated with bisphosphonates, while radiotherapy or orthopedic surgery may be necessary in selected cases. Adjuvant drugs are also often needed [3, 6–8].

In recent years, although new drugs and treatment strategies have substantially improved the median survival (5–8 years) of patients with MMs, the disease remains incurable [9]. In its long trajectory, pain is a frequent occurrence, but there are many other issues to contend with such as cumulative toxicities from the multiple lines of therapy and the physical, psychological, and social aspects of the disease. Thus, the symptom burden is often high and negatively affects quality of life [10–15].

The current approach to the management of MM is to continue treatment for as long as the disease responds [15]. There is now evidence of the importance of establishing a timely and close collaboration between hematologists and PC specialists. Pain, fatigue, anorexia, nausea, depression, and anxiety are the most frequent symptoms of patients with hematologic malignancies. The burden of these symptoms, the long periods of hospitalization, the intensive treatments with significant toxicity, and the uncertain course of the disease are only some of the problems faced by patients. PC has been shown to be beneficial for individuals with solid tumors, and there is now evidence that it could also be useful for hematological malignancies [16, 17].

MM patients can experience years of side effects and symptoms, and within this context, early palliative care (EPC) could help to improve quality of life [15] by managing symptoms more effectively, reducing the need to discontinue specific treatments, and alleviating patient anxiety. It would provide much needed support for caregivers and could help to establish a relationship of trust between patients and PC clinicians, facilitating the development of advance care planning [15].

In Italy, PC services are available for inpatients and outpatients in hematology, oncology, and radiotherapy units in the form of a consultation with PC physician and nurse. For those in poorer clinical conditions or with a higher symptom burden, a PC homecare visit or hospice admission is proposed by a multidisciplinary team (physician, nurse, social worker, psychologist, and physiotherapist).

Within this context, we carried out a retrospective analysis at our cancer institute to evaluate the main reasons for the initial referral of MM inpatients and outpatients to our PC service. We consider our paper a starting point to encourage closer collaboration between hematologists and PC physicians in an effort to find the answers to the many open questions remaining in this area.

## Materials and methods

We carried out a retrospective, single center study on patients with MM. All consecutive MM patients referred by a hematologist for a PC consultation between January 2017 and June 2020 were considered. Patients were identified through our institute’s electronic medical records (CCE Log80 2.6 of Log80 S.r.l.) in which at least one access to the PC unit had been registered. Inclusion criteria were age ≥ 18 years old, MM diagnosis, and ≥ 1 PC consultation at our center.

We considered any setting, i.e., inpatient ward and outpatient clinic. Demographic data (sex, date of birth, date of death), date of MM diagnosis, and date of first consultation with PC team were retrieved. The stage of MM at the first consultation was noted, i.e., diagnosis/first-line treatment, second- or more-line treatment for relapse or progression, and follow-up. We analyzed the setting of the first PC consultation and the reasons behind the referral (presence of symptoms or patient management setting, i.e., hospice or palliative homecare service).

With regard to the clinical aspects, we reviewed the clinical notes in the electronic medical records and focused on the main reasons for the request of a first PC consultation. In particular, we recorded information on the type of pain (neuropathic, somatic, mixed) experienced and on the main cause of the pain (e.g., MM bone lesions, chemotherapy-induced neuropathy, herpes zoster–related neuralgia). We also evaluated the ongoing opioid and adjuvant therapy and any remodulation of the pain therapy needed at the first PC consultation. Other symptoms and needs reported by patients were also taken into consideration. Finally, we analyzed the number of PC consultations required by each patient, and the time lapse between subsequent visits, in particular, the time between the diagnosis and the first visit, the time between the first and other visits, and the time between the last visit and the death of the patient.

Given the descriptive nature of the study, a formal calculation of sample size and statistical power was not performed. Considering a preliminary analysis of clinical medical charts from previous years at our center, it was feasible to include about 40 patients. Descriptive statistical analyses were performed on the entire case series (absolute and relative frequency for categorical variables and means ± standard deviation or median and quantiles for continuous variables). No interim analysis was planned. Institutional informed consent forms for the treatment of personal data had previously been signed by all patients at their first access to our center and included consent to use patient data, materials, and/or diagnostic test results for research purposes. The study was conducted in accordance with the principles laid down in the 1964 Declaration of Helsinki and was approved by the local independent Ethical Committee (Comitato Etico della Romagna, approval no. 8638/2020).

## Results

Of the 325 MM patients followed by our hematologists between January 2017 and June 2020, 43 (13.2%) were referred a PC consultation. Twenty-three (53.3%) were female, and the median age of the entire group was 71 years. Thirty-two (74.4%) patients were evaluated at the outpatient PC clinic, and 11 (25.6%) were seen as inpatients. At the first PC consultation, 20 (46.5%) patients had just been diagnosed or were undergoing first-line treatment, 15 (34.9%) had relapsed, were in progression, or were undergoing second- or more-line treatment, and 8 (18.6%) were in follow-up. At the time of this analysis, 28 (65.1%) patients were alive, and 15 (34.9%) had died.

The main reason for referral to the PC clinic were pain management in 39 (90.7%) patients and evaluation of the most appropriate PC setting (hospice or homecare PC) in 4 (9.3%).

With regard to the 39 MM patients evaluated for pain management, 17 (43.6%) had somatic pain, 9 (23.1%) neuropathic pain, and 13 (33.3%) mixed pain. At the first PC visit, 24 (61.5%) patients had pain from MM bone disease, 6 (15.4%) had chemotherapy-induced neuropathic pain, one (2.6%) had post-herpetic neuralgia, and 8 patients (20.5%) reported pain for other reasons. Table 1 shows the main modifications needed to optimize the pain therapy and the type of opioid prescribed. Four patients were assessed at the PC clinic to identify the most appropriate care setting based on their clinical conditions and needs; 3 were referred for hospice admission, and one was assigned to the palliative homecare service. In the patients initially seen for pain management, 5 subsequently required evaluation of the best PC setting. Three were referred for hospice care and 2 for palliative homecare. A total of 19 (44.2%) patients experienced other symptoms in addition to pain; fatigue in 9 (20.9%), constipation in 8 (18.6%) and depression in 5 (11.6%). Other symptoms often reported at the first PC visit were loss of appetite, cachexia, and insomnia.

**Table 1:** Main modifications to the analgesic therapy and type of opioid prescribed during the first palliative care consultation

	No. patients (%)
Started paracetamol therapy alone	2 (5.1)
Started an opioid therapy	5 (12.8)
Opioid rotation	10 (25.7)
Increased opioid dose	9 (23.1)
Started adjuvant therapy alone	6 (15.4)
Scrambler therapy	2 (5.1)
Opioid rotation + added adjuvant therapy	2 (5.1)
No modified therapy	3 (7.7)
Opioid	
Tramadol	4 (10.2)
Tapentadol	6 (15.4)
Buprenorphine patch	4 (10.2)
Fentanyl patch	7 (18.0)
Oxycodone	9 (23.1)
Morphine sulfate	1 (2.6)
Fentanyl patch + oxycodone	3 (7.7)
Fentanyl patch + morphine sulfate	1 (2.6)
No opioid treatment around the clock	4 (10.2)

The majority of patients had a median of 2 PC consultations (interquartile range 1–4), while only 3 were seen more often by the PC team (9, 13, and 28 PC visits). The median time between MM diagnosis and the first PC consultation was 473 days (interquartile range 57–1099). Details of the time between the first and subsequent PC visits, death, or last follow-up are reported in Table 2. In the 15 patients who died, there was a median of 332 days (interquartile range 60–480) between the first PC visit and death, while the median value (interquartile range 37–401) between the last visit and death was 224 days. The place of death was known for 11 patients; 6 died in a palliative care setting (5 in hospice and one at home followed by the palliative homecare service), 4 in an acute medical unit and one in the hematology ward of our institute.

**Table 2:** Time between diagnosis of multiple myeloma, first and subsequent PC visits, and follow-up or death

	No. patients	Median value (days) (interquartile range)
Diagnosis and 1st PC consultation	43	473 (57–1099)
1st and 2nd PC consultation	27	38 (29–58)
2nd and 3rd PC consultation	14	27 (15–147)
3rd and 4th PC consultation	10	20 (14–29)
4th and 5th PC consultation	5	14 (14–20)
1st and 5th PC consultation	5	112 (73–117)
1st and last PC consultation	27	73 (38–224)
1st PC consultation and death	15	332 (60–480)
1st PC consultation and death/follow-up	43	385 (206–876)
Last PC consultation and death	15	224 (12–875)

*PC*, palliative care

## Discussion

Pain is the most frequent symptom in patients with MM and is almost always present during the course of the disease [3]. The causes of this symptom differ, and the different types of pain generally occur at different stages of the disease. Pain has a dramatic impact on patients, compromising mobility, independence, and quality of life [3, 8]. In agreement with literature data, the majority of the MM patients referred to our PC unit experienced pain, the main causes being MM-related bone disease and chemotherapy-induced peripheral neuropathy. Many of the symptoms were present at diagnosis and at progression/relapse of the disease. As previously stated, the most frequent types of pain were somatic and mixed [2–4, 15]. A large percentage of our study population required opioids, with or without paracetamol, and adjuvant therapy, for pain control.

Given that patients with MM often have severe pain or a difficult type of pain to treat, their management is best suited to a specialized PC team [3, 4, 8, 18]. Furthermore, the rapid development of MM treatment strategies has highlighted the need to carry out more in-depth research into biological issues such as the interactions between opioids and proteasome inhibitors or immunomodulating agents [3]. We believe that a closer collaboration between hematologists and PC specialists could help to answer the numerous open questions remaining in this area. In addition to pain, other symptoms and psychological and social needs often represent a burden for MM patients and their families, sometimes compromising the continuation of MM therapies and negatively affecting quality of life [10–13, 15].

In our study, 19 patients reported other symptoms in addition to pain at the first PC visit, and in around 25% of the population, the PC consultation was aimed at identifying the most appropriate care setting (hospice or palliative homecare service).

Seminal studies have shown that EPC improves symptom management and quality of life in patients with solid tumors [19–21]. Despite strong evidence of the need for PC in patients with hematologist malignancies, EPC is still widely underused in this setting. Consequently, patients tend to be referred for PC late in the course of their disease when they have numerous symptoms that negatively influence quality of life and are often still undergoing intensive treatment [22, 23].

Research has been performed into the potential usefulness of EPC in patients with hematologist malignancies, in particular, those undergoing hematopoietic stem cell transplantation [24–27]. El-Jawahri et al. conducted a randomized clinical trial on patients with acute myeloid leukemia receiving intensive chemotherapy and assigned to integrated palliative and hematology care twice a week during hospitalization or PC on demand. The results revealed an improvement in quality of life, psychological distress, and end-of-life care in patients undergoing the integrated care approach with respect to those receiving on-demand PC [28].

Although the importance of integrating hematology and PC is gaining ground, there is still some hesitancy because of a number of “barriers” such as the unpredictability of the course of the disease; the sudden transition of the disease from stable to progressive; and the sometimes unrealistic expectations of hematologists in relation to antitumor treatments. It is also difficult for the PC team to know when to give active support (e.g., antibiotics or transfusions) to patients who are not in an advanced stage of disease. There is still a limited understanding of PC interventions, a common misconception being that it is only for end-of-life care. Furthermore, the often close bond built up between hematologists and their patients means that it can be difficult to introduce another clinician (especially a PC specialist) into the care scenario [22, 23, 29, 30].

EPC has recently been proposed for use in patients with MM [14, 15]. In this setting, EPC could improve the standard of care during treatment, thus reducing the risk of discontinuation. Early intervention and better symptom management would also help to break down the psychological barriers of patients to PC. Although new models of integration between hematology and PC have been proposed considering different types of hematological diseases and different disease stages, it is clear that they must first be evaluated in studies involving large patient populations [14, 15, 17, 26].

In our study, there was great variability in the timing of patient referral to the PC unit and also in the number of the PC visits carried out for each patient. This was a result of the on-demand rather than routinely scheduled nature of the PC consultations. However, our results are nonetheless in line with those of the literature.

Porta-Sales et al. carried out a retrospective study analyzing, for the first time, the efficacy of EPC in patients with MM (*n* = 67). The median time lapse between MM diagnosis and first PC visit was 355 days, quite early considering that MM has a median survival of 7 years. The authors reported that the early involvement of the PC team led to a rapid reduction in the intensity of physical symptoms, an increase in the administration of opioids for pain, and a subsequent decrease in psychological aspects such as depression and anxiety [14]. They also concluded that an EPC approach would facilitate the decision-making process and, consequently, advance care planning [15, 23].

Our study suggests the usefulness of EPC to manage pain and symptoms in MM patients and to facilitate the decision-making process for the most appropriate end-of-life setting, thus reducing the need for intensive treatments or for access to emergency departments. Not proposing EPC to MM patients could lead to a higher rate of hospitalizations and anti-myeloma therapies in the last months of life, as reported in a retrospective review by Chalopin et al. [31].

The main limitations of our study include its retrospective design and the fact that the data were collected over a long period of time, with suboptimal completeness. Furthermore, the small sample size in a single center setting did not permit any potential associations to be identified. As our study was carried out during the initial period of activity of our PC unit, we evaluated pain and other symptoms exclusively on the basis of information collected in electronic medical records, without the systematic use of validated tools for the identification of the symptoms. Thus, we cannot rule out that some minor symptoms may not have been recorded. Our service has since implemented the use of validated tools for the clinical assessment (i.e., Edmonton Symptom Assessment System, Douleur Neuropathique 4, Memorial Delirium Assessment Scale).

Although a retrospective study methodology is not as strong as that of randomized trial, the findings from our retrospective analysis suggest that EPC could improve the management of MM patients. Further research is warranted in this setting. In conclusion, more randomized clinical trials involving larger populations and multiple centers, together with the standardization of access to EPC for patients with hematological malignancies, especially MM, are needed to improve patient quality of life.

## Data Availability

The datasets generated and/or analyzed during the current study are available from the corresponding author on reasonable request.
